# Myeloid ATP Citrate Lyase Regulates Macrophage Inflammatory Responses *In Vitro* Without Altering Inflammatory Disease Outcomes

**DOI:** 10.3389/fimmu.2021.669920

**Published:** 2021-04-26

**Authors:** Sanne G. S. Verberk, Hendrik J. P. van der Zande, Jeroen Baardman, Kyra E. de Goede, Karl J. Harber, Eelco D. Keuning, Joost M. Lambooij, Frank Otto, Anna Zawistowska-Deniziak, Helga E. de Vries, Menno P. J. de Winther, Bruno Guigas, Jan Van den Bossche

**Affiliations:** ^1^ Department of Molecular Cell Biology and Immunology, Amsterdam Cardiovascular Sciences, Amsterdam Gastroenterology Endocrinology Metabolism, Amsterdam UMC, Vrije Universiteit Amsterdam, Amsterdam, Netherlands; ^2^ Department of Parasitology, Leiden University Medical Center, Leiden, Netherlands; ^3^ Department of Medical Biochemistry, Experimental Vascular Biology, Amsterdam Cardiovascular Sciences, Amsterdam UMC, University of Amsterdam, Amsterdam, Netherlands; ^4^ Witold Stefański Institute of Parasitology, Polish Academy of Sciences, Warsaw, Poland

**Keywords:** obesity, peritonitis, macrophage, immunometabolism, inflammation, ATP citrate lyase

## Abstract

Macrophages are highly plastic, key regulators of inflammation. Deregulation of macrophage activation can lead to excessive inflammation as seen in inflammatory disorders like atherosclerosis, obesity, multiple sclerosis and sepsis. Targeting intracellular metabolism is considered as an approach to reshape deranged macrophage activation and to dampen the progression of inflammatory disorders. ATP citrate lyase (Acly) is a key metabolic enzyme and an important regulator of macrophage activation. Using a macrophage-specific Acly-deficient mouse model, we investigated the role of Acly in macrophages during acute and chronic inflammatory disorders. First, we performed RNA sequencing to demonstrate that Acly-deficient macrophages showed hyperinflammatory gene signatures in response to acute LPS stimulation *in vitro*. Next, we assessed endotoxin-induced peritonitis in myeloid-specific Acly-deficient mice and show that, apart from increased splenic *Il6* expression, systemic and local inflammation were not affected by Acly deficiency. Also during obesity, both chronic low-grade inflammation and whole-body metabolic homeostasis remained largely unaltered in mice with Acly-deficient myeloid cells. Lastly, we show that macrophage-specific Acly deletion did not affect the severity of experimental autoimmune encephalomyelitis (EAE), an experimental model of multiple sclerosis. These results indicate that, despite increasing inflammatory responses *in vitro*, macrophage Acly deficiency does not worsen acute and chronic inflammatory responses *in vivo.* Collectively, our results indicate that caution is warranted in prospective long-term treatments of inflammatory disorders with macrophage-specific Acly inhibitors. Together with our earlier observation that myeloid Acly deletion stabilizes atherosclerotic lesions, our findings highlight that therapeutic targeting of macrophage Acly can be beneficial in some, but not all, inflammatory disorders.

## Introduction

Macrophages are key players in the first line of cellular defense. These highly plastic immune cells can adopt several activation states to respond to the situation at hand. Under homeostatic conditions, functional pro- and anti-inflammatory macrophages are in balance to fight pathogens and to restore tissue damage. Unbalanced macrophage activation may lead to chronic inflammation as seen in atherosclerosis, multiple sclerosis (MS) and obesity or it can lead to hyper-inflammation as occurs during sepsis (1–3).

Metabolic reprogramming of macrophages has been proposed as a promising therapeutic target to combat inflammatory disorders (4, 5). Typical *in vitro* lipopolysaccharide (LPS)-activated inflammatory macrophages switch towards an increased flux through glycolysis and the pentose phosphate pathway to fuel their energy demands for eliciting immune responses, highlighting the central role of metabolism in inflammation (6). Interfering with such metabolic shifts may hamper persistent activation of inflammatory macrophages. Recently, Lauterbach et al. showed that early after LPS activation, the metabolic enzyme ATP citrate lyase (Acly) becomes activated in macrophages and provides the cell with cytosolic acetyl-CoA from increased glucose uptake and citrate accumulation (7). Increased cytosolic acetyl-CoA allows for histone acetylation to stimulate the expression of inflammatory genes and is involved in fatty acid synthesis and cholesterol biosynthesis (7–9). Hereby, Acly links glycolysis and mitochondrial metabolism to lipid metabolism and histone acetylation, marking Acly as a potential metabolic target for tackling excessive inflammation (9, 10). *In vitro* studies indicated that short-term inhibition of Acly by small molecule inhibitors or knockdown through siRNAs can dampen macrophage inflammation (7, 8, 11). Likewise, systemic inhibition of Acly *in vivo* reduces inflammatory outcomes in endotoxin-induced peritonitis (7). However, through a recently developed myeloid-specific Acly knockout mouse model, we revealed a discrepancy in the translation of *in vitro* findings to *in vivo* settings (12). In contradiction to previous studies, LysM-Cre-mediated Acly-deficient macrophages revealed increased inflammatory signaling in *in vitro* LPS-elicited responses and in atherosclerotic plaques *in vivo* (12). Despite increased inflammatory signaling, myeloid Acly deficiency resulted in increased atherosclerotic plaque stability (12). These data underline the need for a better understanding of targeting of Acly in macrophages specifically in the potential treatment of acute and chronic immune disorders.

Here, to decipher how Acly deficiency in myeloid cells affects acute and chronic inflammatory responses, we first analyzed LPS-activated macrophages by RNA-sequencing (RNA-seq). We demonstrated in an unbiased way that Acly-deficient macrophages display deregulated cholesterol handling and elevated inflammatory gene expression signatures after both 3 and 24 hours of LPS stimulation *in vitro*. Remarkably, we show that *in vivo* disease outcomes of different inflammatory conditions, *i.e.* endotoxin-induced peritonitis, obesity and experimental auto-immune encephalitis (EAE) remain largely unaffected by myeloid Acly deficiency. Together with our previous findings in atherosclerosis, these data highlight that myeloid-specific targeting of Acly is beneficial only in some inflammatory disorders and is likely compensated in others.

## Results

### Macrophage Acly Deficiency Increases Inflammatory Signaling *In Vitro*

**Figure 1:**
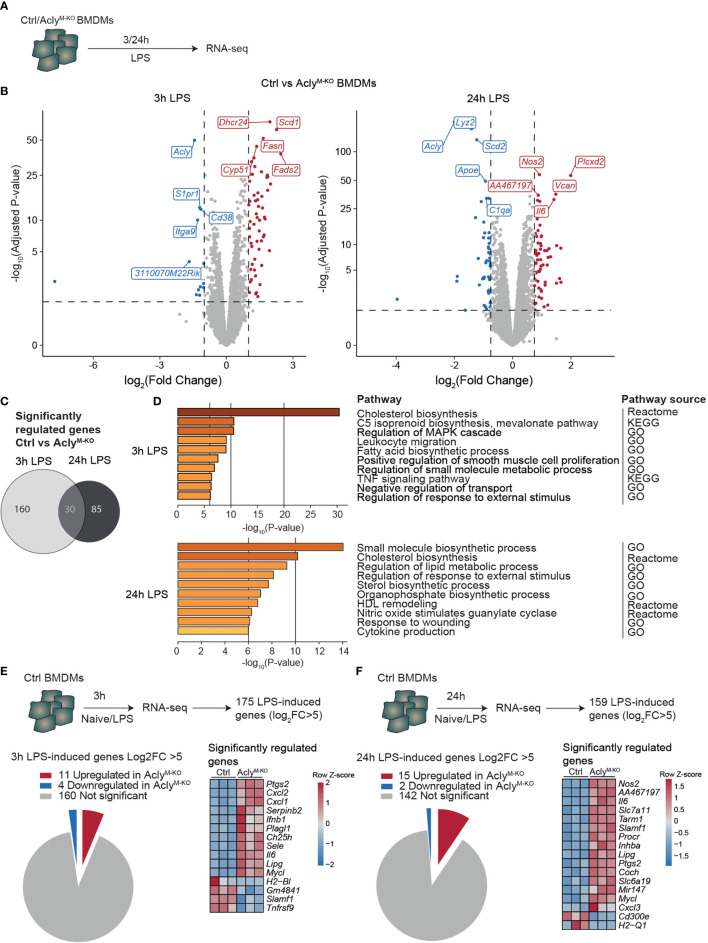
Macrophage Acly deficiency upregulates LPS-induced inflammatory gene expression *in vitro.* **(A)** Control and Acly^M-KO^ BMDMs were stimulated with LPS for 3 or 24 h and analysed by RNA-sequencing. **(B)** Volcano plot of differentially expressed genes between control and Acly-deficient BMDMs after 3 or 24 hour LPS stimulation highlighting the top 5 most significant up- and down-regulated genes. **(C)** Venn diagram showing overlap in deregulated genes by Acly deficiency after 3 and 24 hours LPS-activation **(D)** Deregulated pathways in Acly-deficient macrophages as determined by Reactome, KEGG and GO pathways. **(E, F)** Expression in control versus Acly-deficient BMDMs of genes that are most highly LPS-induced after 3h **(E)** or 24h **(F)** LPS stimulation in control BMDMs. (n=3 per group).

To study the effects of Acly deficiency on inflammatory responses in macrophages, we crossed Acly^fl/fl^ mice with mice expressing Cre under control of the myeloid cell-specific LysM promotor (*Lyz2-Cre*) (12). We have previously shown that naïve, unstimulated Acly-deficient bone marrow-derived macrophages (BMDMs) display deregulated lipid metabolism compared to control BMDMs, whereas inflammatory cytokines were increased only after LPS stimulation (12). Hence, we stimulated control (Acly^fl/fl^) and Acly-deficient (Acly^M-KO^) bone marrow-derived macrophages (BMDMs) *in vitro* with LPS for 3 and 24 hours to examine inflammatory activation in an unbiased way through RNA-seq (**Figure 1A**). Efficiency of *Acly* deletion was confirmed at protein level by Western Blot and at gene expression level by RNA-seq since *Acly* was amongst the most downregulated genes in Acly^M-KO^ macrophages after both 3- and 24-hours LPS stimulation (**Figure 1B**, **Supplementary Figure 1A**). Of all significantly regulated genes between control and Acly^M-KO^ macrophages, only 30 were overlapping after both 3- and 24-hours LPS stimulation, indicating timing-dependent activation patterns (**Figure 1C**). However, pathway analysis revealed that similar pathways were affected by Acly deletion after 3- and 24-hours LPS activation (**Figure 1D**). We found Acly-dependent regulation of pathways related to fatty acid and cholesterol biosynthesis and observed that while cholesterol levels were similar, desmosterol levels were still decreased after inflammatory activation with LPS (**Supplementary Figure 1B**). Next to lipid metabolism, pathway analysis revealed that genes involved in inflammatory signaling were affected upon Acly deletion (**Figure 1D**). To specifically assess the effect of *Acly* deletion in BMDMs on the expression of inflammatory genes, we first selected the most highly induced genes in LPS-stimulated control macrophages at both time points (Log_2_(Fold Change)>5 and adjusted p-value<0.05; **Figures 1E, F**). 15 out of the 175 genes that were most induced after 3 h LPS treatment were differentially expressed in Acly^M-KO^ macrophages in comparison to controls (**Figure 1E**). Likewise, 17 out of the 159 top-induced genes were differentially regulated after 24 h treatment with LPS (**Figure 1F**). At both time points, the majority of differentially expressed genes was upregulated in Acly^M-KO^ macrophages, indicating that deletion of Acly in macrophages potentiates inflammatory responses. Among them, the 5 genes that were significantly altered in naive Acly-deficient BMDMs compared to control BMDMs are mainly involved in lipid metabolism and cell cycle regulation, indicating that increased inflammatory responses are likely not due to differences at baseline (**Supplementary Figure 1C**). *Il6* and *Nos2* were among the most upregulated genes in LPS-stimulated Acly^M-KO^ macrophages, corresponding with our earlier observations that *Acly*-deficient macrophages secrete more IL-6 and nitric oxide in response to LPS (12). Together, this analysis indicates that inflammatory signaling is increased in LPS-activated Acly^M-KO^ macrophages *in vitro*.

### Acute Endotoxin-Induced Peritonitis Is Largely Unaffected in Myeloid-Specific Acly-deficient Mice

**Figure 2:**
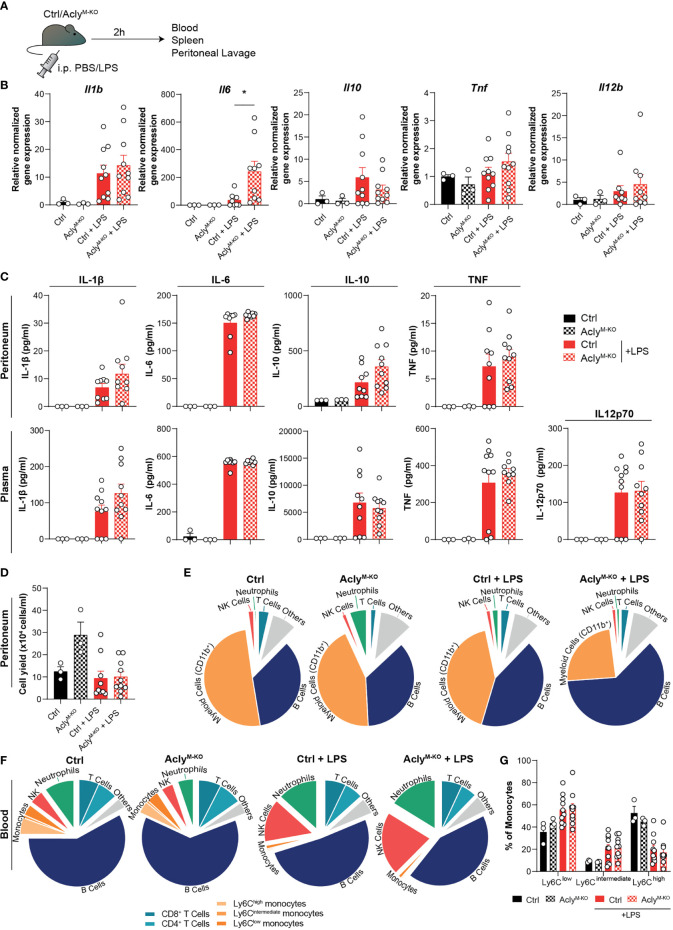
Myeloid Acly deficiency barely alters systemic and local immune responses in acute endotoxin-induced peritonitis. **(A)** Control and Acly^M-KO^ mice received an intraperitoneal injection of LPS or vehicle control. After 2 hours, spleens, blood and peritoneal fluid were collected. **(B)** Splenic gene expression of inflammatory cytokines. **(C)** Peritoneal and plasma cytokine levels in vehicle control and LPS-treated control and Acly^M-KO^ mice. **(D)** Peritoneal exudate cell counts. **(E)** Relative distribution of peritoneal exudate cell levels as assessed by flow cytometry. **(F)** Relative distribution of white blood cells as assessed by flow cytometry. **(G)** Abundance of blood monocyte subsets as defined by Ly6C expression. Values represent mean ± SEM [n=3/3/10/10 (Ctrl vehicle/KO vehicle/Ctrl LPS/KO LPS)]. *P<0.05 by ordinary one-way ANOVA with Sidak’s *post hoc* test for multiple comparisons.

To analyze whether our findings translate to altered acute inflammatory responses *in vivo*, Acly^M-KO^ and control mice were injected intraperitoneally (i.p.) with LPS (or vehicle control) as a commonly used endotoxin-induced peritonitis model (**Figure 2A**) (7, 13). In line with the RNA-seq data, we found increased LPS-induced *Il6* expression in spleens of Acly^M-KO^ mice when compared to control mice, whereas splenic expression of other cytokines remained unaltered (**Figure 2B**). Both local (peritoneal) and systemic (plasma) cytokine and chemokine levels were induced by LPS to a similar extent in Acly^M-KO^ and control mice (**Figure 2C**, **Supplementary Figure 2A**). However, LPS treatment resulted in slightly decreased relative myeloid cell recruitment and slightly increased relative B cell recruitment to the peritoneum in Acly-deficient mice as assessed by flow cytometry on peritoneal exudate cells (**Figures 2D, E**, **Supplementary Figure 2B**). We did not find increased neutrophil-related chemokine gene expression (*Cxcl1*, *Cxcl2*) or tolerogenic cytokine gene expression (*Il10*, *Tgfb*) in peritoneal exudate cells at baseline (**Supplementary Figure 2C**). However, myeloid cells displayed a decreased percentage of viable cells in the peritoneum upon LPS induction, at least partly explaining the reduced abundance of these cells (**Supplementary Figure 2D**). To investigate whether circulating immune cell levels were altered in the absence of myeloid Acly, we analyzed blood leukocytes. Flow cytometry revealed increased frequencies of NK cells and neutrophils in response to LPS injection, with no differences between control and Acly^M-KO^ mice (**Figure 2F**). Interestingly, circulating neutrophils in Acly^M-KO^ mice displayed also decreased viability in response to LPS, indicating regulation of cell survival by Acly (**Supplementary Figure 2E**). Circulating monocyte subtypes displayed a similar increase in Ly6C^low^ and Ly6C^int^ abundance at the expense of Ly6C^high^ upon LPS injection in both control and Acly^M-KO^ mice (**Figure 2G**). Together, these data indicate that myeloid Acly deficiency alters neither local nor systemic cytokine responses and results in slightly altered cellular responses upon LPS injection locally, potentially through regulation of myeloid cell viability.

### Obesity-Induced Chronic Low-Grade Inflammation and EAE Onset and Severity Are Unaffected in Myeloid-Specific Acly-Deficient Mice

**Figure 3:**
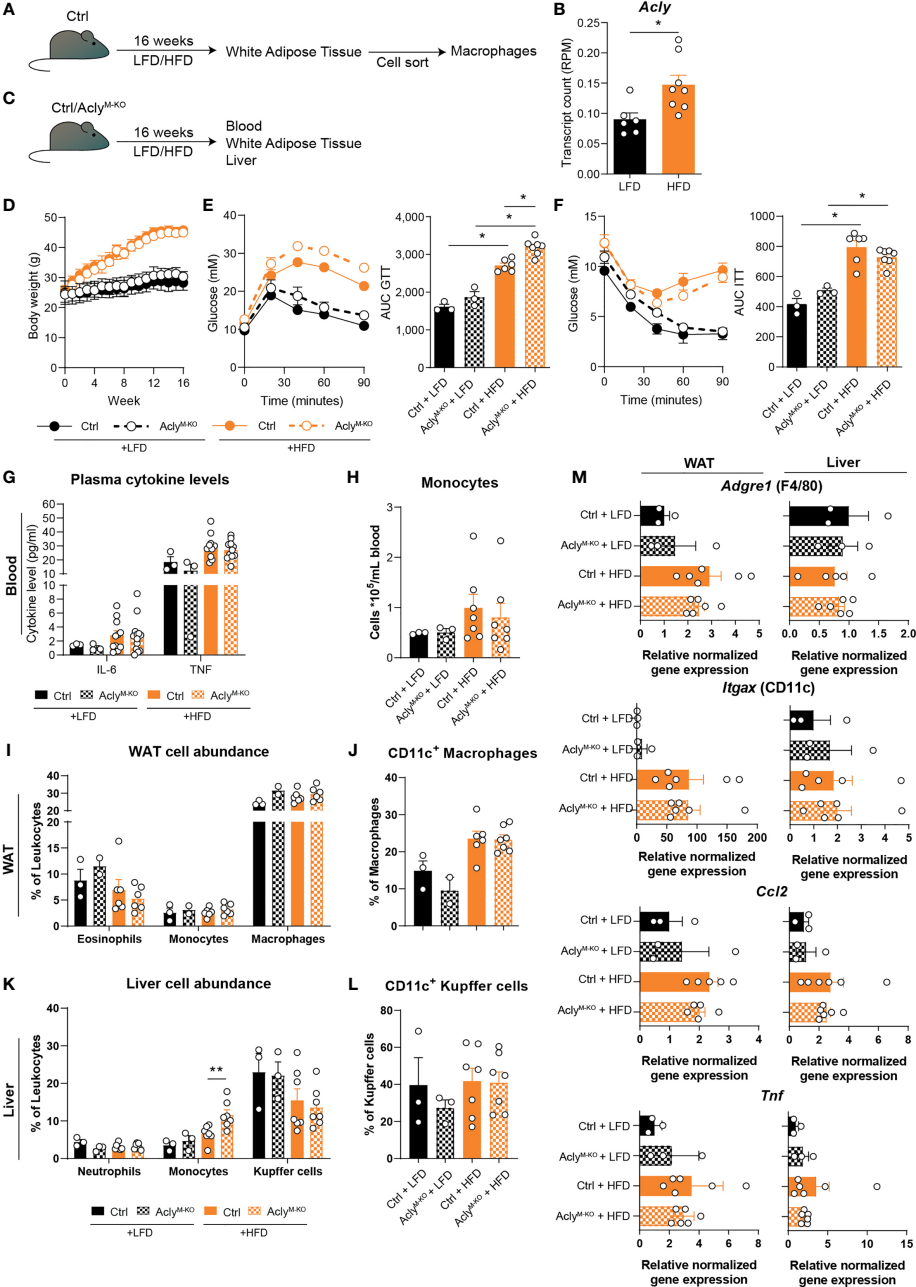
Obesity-related chronic low-grade inflammation remains unaffected by myeloid Acly deficiency. **(A)** Control mice received a control low-fat diet (LFD) or a high-fat diet (HFD) for 16 weeks, after which macrophages were sorted from white adipose tissue (WAT). **(B)** *Acly* expression in WAT macrophages after 16 weeks of LFD or HFD. [n=6/8 (LFD/HFD)]. **(C)** Control and Acly^M-KO^ received a LFD or HFD for 16 weeks, after which blood, liver and WAT were collected. **(D)** Body weight during the course of 16 weeks. **(E)** Blood glucose levels during glucose tolerance test (GTT) and derived area under the curve (AUC) in control and Acly^M-KO^ mice after LFD or HFD. **(F)** Blood glucose levels during insulin tolerance test (ITT) and AUC in control and Acly^M-KO^ mice after LFD or HFD [n=3/3/6/7 (Ctrl LFD/KO LFD/Ctrl HFD/KO HFD)]. **(G)** Plasma cytokine levels at 12 weeks of diet intervention [n=3/3/10/10 (Ctrl LFD/KO LFD/Ctrl HFD/KO HFD)]. **(H)** Circulating monocyte levels. **(I)** WAT myeloid cell abundance, **(J)** percentage of CD11c^+^ macrophages in WAT. **(K)** Liver myeloid cell abundance, **(L)** percentage of CD11c^+^ Kupffer cells. **(M)** Gene expression levels of indicated genes in liver and WAT [n=3/3/6/7 (Ctrl LFD/KO LFD/Ctrl HFD/KO HFD)]. Values represent mean ± SEM. *P<0.05, **P<0.01 by ordinary one-way ANOVA with Sidak’s *post hoc* for multiple comparisons.

Obesity and MS are diseases characterized by chronic immune activation, which drives disease progression. During obesity, adipose tissue macrophages (ATMs) are exposed to a lipid-rich environment that drives pro-inflammatory macrophage activation (14). To assess the effect of obesity on Acly levels in ATMs, we assessed its expression in an RNA-seq data set from ATMs that were sorted from mice fed a control low-fat diet (LFD) or a high-fat diet (HFD) for 16 weeks (**Figure 3A**). Obesity increased *Acly* expression in ATMs from obese mice (**Figure 3B**), which led us to investigate the effects of myeloid Acly deficiency on metabolic outcomes in obesity. Hence, we fed control and Acly^M-KO^ mice a LFD or HFD for 16 weeks and assessed whole-body metabolic parameters and chronic low-grade inflammation in the circulation and metabolic tissues (**Figure 3C**). Myeloid-specific Acly deficiency did not impact diet-induced body weight changes when compared to controls (**Figure 3D**). However, Acly^M-KO^ mice displayed slightly aggravated glucose intolerance after HFD feeding (**Figure 3E**), whereas insulin sensitivity was unaffected (**Figure 3F**). As expected, plasma IL-6 and TNF levels and circulating monocytes were higher in HFD-fed mice, but were not affected by myeloid-specific Acly deficiency (**Figures 3G, H**, **Supplementary Figure 3A**). In both visceral white adipose tissue (epididymal; eWAT) and the liver, two of the main metabolic organs, diet-induced changes in myeloid cell composition detoriate tissue-specific insulin sensitivity. In WAT, depletion of eosinophils, recruitment of monocytes and accumulation of neutrophils and CD11c^+^ pro-inflammatory macrophages are associated with metabolic dysfunctions (15). While HFD feeding indeed increased CD11c^+^ macrophage abundance, myeloid Acly deficiency did not alter these changes (**Figures 3I, J**, **Supplementary Figures 3B, C**). Also, neutrophil abundance in WAT was similar after HFD feeding in both genotypes (**Supplementary Figure 3D**). Likewise, recruitment of neutrophils, monocytes and activation of Kupffer cells are associated with hepatic insulin resistance (16–18). Whereas hepatic neutrophil recruitment was unchanged, Kupffer cells were increasingly activated upon HFD feeding. Acly deficiency did not affect these parameters, but did result in increased hepatic monocyte recruitment upon HFD feeding when compared to HFD fed controls. (**Figures 3K, L**, **Supplementary Figure 3B**). Yet, gene expression of typical obesity-induced macrophage and cytokine genes *Adgre* (F4/80)*, Itgax* (CD11c), *Tnf*, and *Ccl2* remained unaltered by myeloid Acly deficiency in both eWAT and liver (**Figure 3M**). These data indicate that obesity-induced chronic low-grade inflammation remains unaffected upon myeloid Acly deficiency *in vivo* and causes minor local changes. Lastly, during MS, chronic inflammatory activation affects the central nervous system. We analyzed the effect of myeloid Acly deficiency on disease onset and progression by applying an EAE model (**Supplementary Figure 4A**). Also in this chronic inflammatory condition, we did not find differences in disease onset and severity between control and Acly^M-KO^ mice (**Supplementary Figures 4B–E**). Taken together, our data indicate that deleting Acly in macrophages increased their inflammatory potential *in vitro*, but did not affect acute and chronic inflammatory conditions in peritonitis, obesity and EAE *in vivo*.

## Discussion

In recent years, Acly arose as a potential metabolic target in combating inflammatory disorders (7, 8, 11, 12). In this study, we demonstrated that Acly-deficient BMDMs have a hyperinflammatory gene signature when activated with LPS *in vitro*. Apart from increased splenic *Il6* expression, other systemic or local inflammatory readouts determined in blood, spleen, and peritoneum were unaltered in myeloid-specific Acly-deficient mice during endotoxin-induced peritonitis *in vivo*. Likewise, neither obesity nor EAE-related inflammation were affected by myeloid Acly deficiency. These results indicate that, although *in vitro* inflammatory responses were increased in LysM-Cre-mediated Acly-deficient cells, myeloid Acly deficiency did not alter acute and chronic inflammatory disease outcomes *in vivo* in mouse models of obesity, peritonitis, and MS. These results highlight that therapeutic targeting of macrophage Acly is likely not beneficial for all inflammatory disorders.

Targeting Acly in inflammation provides the opportunity to target cellular respiration, cholesterol biosynthesis, and histone acetylation (9). In an earlier study, we observed similar cellular respiration and glycolysis rates, alike histone acetylation levels but a disrupted fatty acid and cholesterol biosynthesis in naïve Acly-deficient macrophages (12). Our current observations confirm that even after 3- or 24-hour LPS-activation, cholesterol biosynthesis and related pathways are still among the most deregulated pathways in Acly-deficient macrophages, indicating the persistence of the effect of Acly knockdown in macrophages. Interestingly, RNA-sequencing of BMDMs treated with an Acly inhibitor in combination with LPS for 4 hours does not show a deregulation of cholesterol metabolism, but rather highlights pathways related to immune responses (7). These discrepancies may be explained by differences in the timing of the Acly inhibition, knockdown and knockout methods. Whereas acute inhibition or siRNA-mediated knockdown might not allow sufficient time for the cell to overcome acute metabolic dysregulation, a genetic knockout approach can induce adaptive changes to rewire metabolic pathways during differentiation and hereby secure sufficient cytosolic acetyl-CoA levels. Indeed, genetic Acly deficiency in multiple mammalian cell types shows an increased abundance of Acetyl-CoA synthetase short chain family member 2 (ACCS2) as well as an increased flux through this enzyme to secure cytosolic acetyl-CoA for *de novo* lipogenesis from endogenous acetate (19–21). In turn, this acetate-derived acetyl-CoA in cancer cells can induce histone acetylation and lipogenesis (22). In line with this, both unstimulated and 3-hour LPS-activated Acly-deficient macrophages display increased expression of *Acss2*, indicating that macrophages can at least partly restore acetyl-CoA levels and adapt metabolism upon long-term Acly deletion (12).

Apart from differences in regulation of metabolism between genetic *Acly* deletion and acute inhibition *in vitro*, both methods of targeting Acly show opposite regulation of inflammatory responses (7, 8, 11). Since metabolism can dictate inflammatory responses in macrophages, the hyperinflammatory response in Acly-deficient macrophages may be an effect of i) increased flux through ACSS2 and/or ii) deregulated cholesterol biosynthesis. Firstly, in line with our results, Acly-deficient adipocytes show increased inflammatory gene signaling in combination with increased *Acss2* expression (21). Also T cells with affected metabolism and an increased flux through ACSS2 have been shown to display an augmented inflammatory response (23). These findings fit with the notion that acetate-derived acetyl-CoA is able to drive increased inflammatory responses (24). The possible link between ACSS2 and inflammation is a potential mechanistic explanation that increased ACSS2 may, at least partly, be a determinant of increased inflammatory signaling during genetic *Acly* deficiency. Secondly, we previously showed in unstimulated Acly-deficient macrophages that a deregulation of cholesterol biosynthesis results in decreased levels of the cholesterol pathway-intermediate desmosterol (12). Decreased desmosterol levels and subsequent blunted inhibition of inflammatory responses by liver X receptor (LXR) may also partly explain the hyperinflammatory genotype of Acly-deficient inflammatory macrophages. Since desmosterol levels are still decreased after 3- and 24-hour LPS stimulation, it highlights that cholesterol-related pathways are still affected on both gene expression and sterol level in LPS-stimulated Acly-deficient macrophages. When such metabolic adaptation occurs during long-term inhibition with small molecule inhibitors of Acly remains unknown and could be examined by using conditional inducible knockouts.

We now demonstrate that genetic *Acly* deletion specifically in macrophages does not alter inflammatory readouts in endotoxin-induced peritonitis except for splenic *Il6* expression, indicating that myeloid Acly deficiency may only mildly alter local LPS-elicited inflammatory responses in the spleen. Splenic *Il6* is expressed by bone marrow-derived myeloid cells upon i.p. LPS injection (25), which indicates that increased splenic *Il6* expression in myeloid Acly-deficient mice is likely due to its higher expression by myeloid cells. However, since only local alterations were evident in myeloid Acly-deficient mice, it implies that there are unidentified factors that explain the lack of a hyperinflammatory response *in vivo*, which we did observe *in vitro*. While expression of tolerogenic cytokines or chemo-attractant chemokines in peritoneal cells was similar in control and Acly^M-KO^ at baseline, there could be other unanalyzed factors regulated by myeloid Acly at baseline that attenuate a hyperinflammatory response *in vivo*. Another explanation of differences in responses *in vitro* and *in vivo* could be that tissue-resident macrophages do not all express equal amounts of Acly or the LysM-promotor at baseline or after activation, indicating that myeloid Acly deletion mediated by the LysM-Cre system will not affect all tissues to a similar extent (as can be extracted from GSE63341, GSE122108). Alternatively, decreased viability of myeloid cells upon i.p. injection with LPS might explain a reduced ability to mount inflammatory responses *in vivo*.

In sharp contrast, systemic inhibition of Acly *in vivo* with small molecules resulted in lower circulating cytokine levels in an endotoxin-induced model of peritonitis (7). This implies that beneficial effects from systemic inhibition with a small molecule inhibitor are either not solely mediated by its effect on macrophages, or cells undergoing small molecule-inhibition of Acly are not successive to adaptive metabolic changes as Acly-deficient macrophages are. However, since small molecule inhibition in macrophages *in vitro* shows decreased inflammation, it is likely that beneficial effects in peritonitis are at least partly macrophage-mediated.

Interestingly, Acly inhibition was also shown recently to be utilized for immune evasion by uropathogenic *Escherichia coli* by suppressing cytokine production during cystitis, providing additional evidence that Acly is linked to inflammatory responses (26). Additionally, circulating Acly was recently shown to be increased during sepsis, suggesting an immunological role for Acly in the disease (27). We found a decreased abundance of viable myeloid cells after activation with LPS in Acly^M-KO^ mice and an increased abundance of neutrophils at baseline, which might indicate that responses to an infection with live pathogen may be altered upon myeloid Acly deficiency. Although sterile, acute LPS-induced peritonitis does not fully reflect bacterial infections, common transcriptional macrophage responses to both gram-positive and –negative bacteria and bacterial compounds have been shown (28). Together with our findings, one could speculate that Acly^M-KO^ mice respond similar to live pathogen challenge when compared to control mice. However, this should be examined in more detail in future studies.

Lastly, we show that WAT macrophages display increased expression of *Acly* after HFD feeding. Genetic myeloid *Acly*-deletion, in turn, did not alter inflammatory outcomes, but resulted in a slightly impaired glucose tolerance in obesity. As whole-body insulin sensitivity was not impaired, this hints at a mild inhibition of insulin-independent glucose uptake. Interestingly, obese mice with an adipocyte-specific knockout of *Acly* also showed impaired glucose handling (21). Additionally, Acly expression is positively correlated with glucose transporter 4 (GLUT4) expression in human adipose tissue (21), indicating co-regulation of these proteins in this particular setting. If such co-regulation of glucose transporters exists, one cannot completely exclude the possibility that the observed metabolic defect in Acly^M-KO^ mice is secondary to decreased glucose uptake in myeloid cells.

Collectively, our results indicate that caution is warranted in prospective long-term or chronic treatments of inflammatory disorders with macrophage-specific Acly inhibitors. The findings of this study aid in further understanding the interaction between macrophage Acly and inflammatory disorders. Further studies into the development of new cell-specific Acly inhibitors can build upon the idea that chronic myeloid Acly therapy does not benefit or worsen inflammatory disorders like sepsis, obesity and EAE.

## Methods

### Animals, Treatment and Diet

C57Bl/6J mice with *loxP* sites flanking exon 9 of the *Acly* gene (*Acly^f^* ^l/fl^) (20) were crossed with *Lyz2*-Cre transgenic mice to generate mice with a myeloid-specific deletion of *Acly* (Acly^M-KO^). All mouse experiments were conducted after approval by the Committee for Animal Welfare (University of Amsterdam, VU University Amsterdam and Leiden University Medical Center).

Acute endotoxin-induced peritonitis was achieved by intraperitoneally injecting age-, weight-, and sex-matched 17 week old control and Acly^M-KO^ mice with 5 µg/g bodyweight LPS (From Escherichia coli serotype O55:B5; Sigma) in PBS or with PBS only for control. Mice were randomly allocated to either PBS control (n=3) or LPS experimental conditions (n=10). 2 hours after LPS injection, mice were euthanized by CO_2_ asphyxiation. Blood was collected by cardiac puncture with ethylene-diamine-tetraacetatic acid (EDTA; Gibco)-pretreated syringes. Peritoneal lavage was performed by injecting 5 mL 2mM EDTA in PBS in the peritoneal cavity followed by careful removal of the maximum volume of lavage fluid possible (3.8-4.6 mL). Subsequently, spleens were harvested and snap frozen in liquid nitrogen for RNA isolation.

EAE was induced in age- and sex-matched 12 week-old control and Acly^M-KO^ mice by 0.2 mL subcutaneous injection of myelin oligodendrocyte glycopeptide (MOG)_35-55_ in an emulsion with complete Freund’s adjuvant (CFA; Hooke Laboratories) followed by two times intraperitoneal injection of 200 ng pertussis toxin (PTX) dissolved in PBS (Hooke Laboratories) on 2 successive days. Mice were weighed, monitored and scored on EAE symptoms (0 = healthy; 1 = limp tail; 2 = ataxia and/or paresis of hind limbs; 3 = paralysis of hind limbs and/or paresis of forelimbs; 4= tetraplegia; 5 = moribund or dead) (29, 30) daily for the course of 30 days by 2 independent researchers blinded to mouse genotypes. In case clinical signs were less severe than typically observed, clinical scores were graded as ‘x-0.5’.

Obesity was induced by feeding mice a high-fat diet (HFD). Group randomization was systematically performed before the start of the experiment, based on age, body weight, fat mass and fasting blood glucose levels. 9 to 17 week old male control and Acly^M-KO^ mice were fed a low-fat diet (LFD, 10 kcal% fat, D12450B, Research Diets) (n=4) or a high-fat diet (45 kcal% fat, D12451, Research Diets) (n=8) for 16 weeks, during which body weight was monitored using a conventional weighing scale.

### BMDM Isolation and BMDM Culture

Bone marrow cells were flushed from femurs and tibias of control and Acly^M-KO^ mice. Bone marrow-derived macrophages (BMDMs) were generated by culturing in complete RPMI-1640 (Gibco) containing 25 mM HEPES, 2 mM L-glutamine, 10% FCS (Gibco), 100 U/ml penicillin, 100 µg/ml streptomycin (Gibco), and 15% L929-conditioned medium (LCM) for 7 days. Control and Acly^M-KO^ cells were collected and plated for RNA-sequencing at a density of 5*10^5^ cells per well in a 24-well plate and left untreated or stimulated with 100 ng/mL LPS (Sigma) for 3 or 24 hours.

### Transcriptomics

Total RNA was isolated from BMDMs using an RNeasy Mini Kit with DNase treatment (QIAGEN) followed by strand-specific library construction using the KAPA mRNA HyperPrep kit (KAPA Biosystems). Samples were sequenced as previously described (12, 31). Briefly, sequencing was performed on an HiSeq 4000 instrument (Illumina). Reads were aligned to mouse genome mm10 using *STAR 2.5.2b*. Indexing and filtering of BAM files was done with *SAMtools* after which raw tag counts and RPKM values were summed using HOMER2’s AnalyzeRepeats.pl script. Differentially expressed genes were analyzed using *DESeq2* package in R. Volcano plots and heatmaps were generated using *ggplot2, ggrepel* and *pheatmap* packages. Genes were considered differentially expressed between control and Acly^M-KO^ BMDMs when Log2 fold change >0.75 and adjusted p-value <0.05. Pathway analysis was performed by Metascape (32) [http://metascape.org] on regulated genes in 3 or 24 hour LPS-induced Acly^M-KO^ BMDMs when compared to 3 or 24 hour LPS-induced control BMDMs, respectively.

### Immunoblotting

Immunoblotting was performed as described previously (12). Briefly, BMDMs stimulated with LPS for 24 hours and lysed on ice in NP40 cell lysis buffer (ThermoFisher) with fresh protease inhibitor cocktail (Sigma-Aldrich) and fresh PhosSTOP (Sigma-Aldrich). Lysates were analyzed for protein concentration with a BCA assay (ThermoFisher) and inactivated by heating at 95°C for 10 min. 4-12% Bis-Tris gels (ThermoFisher) were used for protein separation and nitrocellulose membranes (Bio-Rad) for blotting. Membranes were incubated with antibodies against ACLY (1:1000, Abcam, ab40793) and α-Tubulin (1:2000, Sigma-Aldrich, T5168) and signal was visualized using horseradish peroxidase (HRP)-conjugated secondary antibodies in 5% BSA TBS-T and developed using SuperSignal West Pico Chemiluminescent PLUS Substrate (ThermoFisher).

### Sterol Analysis

Sterol analysis in LPS-activated macrophages was performed as described previously (12). Briefly, LPS-stimulated BMDMs were homogenized and incubated with an internal standard and saponificated during 2-hour incubation at 80°C. Sterols were extracted using hexane and quantified using a GC system (CPSil5 column, Agilent GC 7890B) with FID detection followed by GC-MS and selected ion monitoring of tMS-derivaties on an MSD5977A MS detector in EI+-mode.

### Gene Expression Analysis

Total RNA from snap frozen spleens was isolated using GeneJET RNA Purification Kit from ThermoFisher and following manufacturer’s protocol for Total RNA Purification from Mammalian Tissue. Briefly, tissue was disrupted by crushing with mortar and pestle in lysis buffer supplemented with 2% v/v β-mercaptoethanol. Lysates were homogenized by pipetting up and down and transferred to tubes before vortexing. Subsequently, samples were deproteinized by proteinase K. RNA isolation was performed on supernatants after centrifugation using the Purification Columns and wash buffers provided with the isolation kit. cDNA was transcribed using a High-Capacity cDNA Reverse Transcription Kit (ThermoFisher). Gene expression analysis was performed with SYBR Green Fast mix (Applied Biosystems) on a Viia7 system (Applied Biosystems). Expression levels were normalized to average levels of housekeeping genes ribosomal protein large P0 (*Rplp0*) and Cyclophilin A (*Ppia*).

RNA from snap-frozen adipose tissue and liver samples of LFD and HFD-fed mice was isolated using Tripure RNA Isolation reagent (Roche Diagnostics) and the phenol-chloroform extraction method. Total RNA (1 µg) was reverse transcribed and quantitative real-time PCR was performed with SYBR Green Core Kit on a MyIQ thermal cycler (Bio-Rad). mRNA expression was normalized to *RplP0* mRNA content and expressed as fold change compared to LFD-fed control mice as indicated, using the ΔΔCT method. Primer sequences used are depicted in **Supplementary Table 2**.

### Cytokine and Chemokine Analysis

Levels of IL-6, TNF, IL-12p70, IL-1β, and IL-10 were quantified in plasma and peritoneal lavage from mice with acute LPS-induced peritonitis using ELISA (Life Technologies), according to manufacturer’s protocol. CXCL1 and CXCL2 were analyzed in peritoneal lavage fluid from naïve and LPS-injected mice using ELISA (R&D systems), according to manufacturer’s protocol. Circulating IL-6 and TNF levels in obese mice were analyzed on plasma samples from 4h-fasted mice using the Cytometric Bead Array enhanced sensitivity kits (CBA; BD Biosciences) according to the manufacturer’s recommendations.

### Isolation of Stromal Vascular Fraction From Adipose Tissue

After 16 weeks on diet, LFD-fed lean and HFD-fed obese mice were sacrificed through an overdose of ketamine/xylazine. eWAT was collected after a 1 minute transcardial perfusion with PBS and digested as described previously (33, 34). In short, collected tissues were minced and incubated for 1 hour at 37°C under agitation (60 rpm) in HEPES-buffered Krebs solution (pH 7.4) containing 0.5 g/L collagenase type I from *Clostridium histolyticum* (Sigma-Aldrich), 2% (w/v) dialyzed bovine serum albumin (BSA, fraction V; Sigma-Aldrich) and 6 mM D-Glucose. The disaggregated adipose tissue was passed through a 200 μm filter (Sefar) that was washed with PBS supplemented with 2.5 mM EDTA and 1% FCS. After allowing the adipocytes to settle, the infranatant, consisting of immune cells, was collected and pelleted at 350 x g for 10 minutes at room temperature. Subsequently, the pellet was treated with erythrocyte lysis buffer (0.15 M NH_4_Cl; 1 mM KHCO_3_; 0.1 mM Na_2_EDTA). Cells were next washed with PBS/EDTA/FCS, and counted using a hemocytometer.

### Isolation of Leukocytes From Liver Tissue

Livers were collected and digested as described previously (33, 34). In short, livers were minced and incubated for 45 minutes at 37°C in RPMI 1640 + Glutamax (Life Technologies) containing 1 mg/mL collagenase type IV from *C. histolyticum*, 2000 U/mL DNase (both Sigma-Aldrich) and 1 mM CaCl_2_. The digested liver tissues were passed through a 100 μm cell strainer that was washed with PBS/EDTA/FCS. Following washing with PBS/EDTA/FCS, samples were centrifuged at 50 x g to pellet hepatocytes (3 minutes at 4°C). Next, supernatants were collected and pelleted (530 x g, 10 minutes at 4°C). Following erythrocyte lysis, CD45^+^ leukocytes were isolated using LS columns and CD45 MicroBeads (35 µL beads per liver, Miltenyi Biotec) according to manufacturer’s protocol and counted using a hemocytometer.

### Flow Cytometry

White blood cells from mice with acute endotoxin-induced peritonitis were collected by centrifugation of 1 mL collected blood and subsequent red blood cell lysis with ACK lysis buffer. Cells from peritoneal lavage were collected by centrifugation. White blood cells and cells collected by peritoneal lavage were labeled by staining for 30 minutes on ice in the dark with the following fluorescently-labelled antibodies diluted in staining buffer: CD8-BV421 (1:100), CD4-BV510 (1:150), Ly6C-BV605 (1:600), CD11c-BV650 (1:100), F4/80-BV711 (1:100), CD45-BV785 (1:500), Ly6G-FITC (1:200), MHC-II-PerCP-Cy5.5 (1:400), CD19-PE (1:100), CD11b-PE-Cy7 (1:400), NK1.1-APC (1:200), CD3-AF700 (1:50) (all from Biolegend). Unspecific antibody binding was blocked by an anti-CD16/32 antibody (1:100, BD Bioscience) and dead cells were excluded from analysis after staining with fixable viability dye-e780 (1:1000, eBioscience). Fluorescence was captured using a BD LSR Fortessa and analyzed using FlowJo 10.0.7 analysis software.

Purified epidydimal white adipose tissue (eWAT) stromal vascular cells and liver leukocytes were stained with the fixable live/dead marker Zombie-UV (1:1000; Invitrogen), fixed with 1.9% formaldehyde (Sigma-Aldrich) and stored in staining buffer at 4°C in the dark until subsequent surface staining and flow cytometry within 4 days. Cells were labeled with the following fluorescently-labelled antibodies diluted in staining buffer: Siglec-F-BV605 (1:200; BD Biosciences), CD64-PE (1:100), Ly6C-APC-Cy7 (1:700), CD11c-BV421 (1:100), F4/80-BV711 (1:200), CD45-BV785 (1:400; all Biolegend) and CD11b-PE-Cy7 (1:6000; eBioscience), for analysis of innate immune cells, and CD11c-FITC (1:100), CD11b-FITC (1:100), GR-1-FITC (1:200), CD4-BV650 (1:200; all BD Biosciences), NK1.1-FITC (1:400; eBioscience), B220-PE-Cy7 (1:200), CD3-BV605 (1:400), CD8-BV711 (1:200) and CD45-BV785 (1:400; all Biolegend) for analysis of adaptive immune cells.

All antibodies used for flow cytometry are listed in **Supplementary Table 3**.

### Glucose and Insulin Tolerance Tests

An intraperitoneal whole-body glucose tolerance test (ipGTT) was performed after 15 weeks on diet in 6h-fasted mice, as previously reported (33, 34). In short, after an initial blood collection from the tail vein (t = 0), a glucose load (2 g/kg total body weight of D-Glucose; Sigma-Aldrich) was administered i.p., and blood glucose was measured at 20, 40, 60, and 90 min after glucose administration using a hand-held Glucometer (Accu-Chek).

An intraperitoneal whole-body insulin tolerance test (ipITT) was performed after 15 weeks on diet in 4h-fasted mice, as described previously (33, 34). Briefly, a bolus of insulin (0.75U/kg total body mass; NOVORAPID) was administered i.p. after an initial blood collection from the tail vein (t = 0), and blood glucose was measured at 20, 40, 60, and 90 min after insulin administration using a Glucometer.

### Statistical Analysis

Data are presented as mean ± standard error of the mean (SEM). Statistical significance was tested using either a two-tailed Student’s *t* test for comparing 2 samples or an ordinary one-way ANOVA followed by Sidak’s correction for multiple comparisons in GraphPad Prism software (8.2.1). P-values < 0.05 were considered statistically significant indicated by *p<0.05, **p<0.01, ***p<0.001.

## Data Availability Statement

The datasets presented in this study can be found in online repositories. The names of the repository/repositories and accession number(s) can be found below: NCBI Gene Expression Omnibus, accession no: GSE169189.

## Ethics Statement

The animal study was reviewed and approved by Committee for Animal Welfare (University of Amsterdam, VU University Amsterdam and Leiden University Medical Center).

## Author Contributions

SV and HZ contributed equally to this work. Conceptualization: JVB and BG. Methodology: SV, HZ, JB, and KG. Formal analysis: SV and HZ. Investigation: SV, HZ, JB, KG, KH, EK, JL, FO, and AZ-D. Visualization: SV. Writing-review & editing: SV, HZ, HV, MW, BG, and JVB. Funding acquisition: JVB, HZ, BG and MW. All authors contributed to the article and approved the submitted version.

## Funding

JVB received a VENI grant from ZonMW (91615052) and a Netherlands Heart Foundation junior postdoctoral grant (2013T003) and senior fellowship (2017T048). MW is an established investigator of the Netherlands Heart Foundation, is supported by grants from the Netherlands Heart Foundation and Spark-Holding BV (2015B002 and 2019B016), and Fondation Leducq (16CVD-01), and holds an AMC fellowship. BG received a ZonMW TOP grant from NWO, ZonMW (91214131) and HZ received a grant supported by the NWO Graduate School Program (022.006.010).

## Conflict of Interest

The authors declare that the research was conducted in the absence of any commercial or financial relationships that could be construed as a potential conflict of interest.

## References

[1] Osuchowski MF Welch K Siddiqui J Remick DG. Circulating Cytokine/Inhibitor Profiles Reshape the Understanding of the SIRS/CARS Continuum in Sepsis and Predict Mortality. J Immunol (2006) 177(3):1967–74. 10.4049/jimmunol.177.3.1967 16849510

[2] Moore KJ Sheedy FJ Fisher EA. Macrophages in Atherosclerosis: A Dynamic Balance. Nat Rev Immunol (2013) 13(10):709–21. 10.1038/nri3520 PMC4357520 23995626

[3] Chawla A Nguyen KD Goh YP. Macrophage-Mediated Inflammation in Metabolic Disease. Nat Rev Immunol (2011) 11(11):738–49. 10.1038/nri3071 PMC3383854 21984069

[4] O’Neill LA Pearce EJ. Immunometabolism Governs Dendritic Cell and Macrophage Function. J Exp Med (2016) 213(1):15–23. 10.1084/jem.20151570 26694970 PMC4710204

[5] Van den Bossche J O’Neill LA Menon D. Macrophage Immunometabolism: Where are We (Going)? Trends Immunol (2017) 38(6):395–406. 10.1016/j.it.2017.03.001 28396078

[6] Jha AK Huang SC Sergushichev A Lampropoulou V Ivanova Y Loginicheva E. Network Integration of Parallel Metabolic and Transcriptional Data Reveals Metabolic Modules That Regulate Macrophage Polarization. Immunity (2015) 42(3):419–30. 10.1016/j.immuni.2015.02.005 25786174

[7] Lauterbach MA Hanke JE Serefidou M Mangan MSJ Kolbe CC Hess T. Toll-Like Receptor Signaling Rewires Macrophage Metabolism and Promotes Histone Acetylation Via ATP-Citrate Lyase. Immunity (2019) 51(6):997–1011 e7. 10.1016/j.immuni.2019.11.009 31851905

[8] Langston PK Nambu A Jung J Shibata M Aksoylar HI Lei J. Glycerol Phosphate Shuttle Enzyme GPD2 Regulates Macrophage Inflammatory Responses. Nat Immunol (2019) 20(9):1186–95. 10.1038/s41590-019-0453-7 PMC6707851 31384058

[9] Wellen KE Hatzivassiliou G Sachdeva UM Bui TV Cross JR Thompson CB. ATP-Citrate Lyase Links Cellular Metabolism to Histone Acetylation. Science (2009) 324(5930):1076–80. 10.1126/science.1164097 PMC2746744 19461003

[10] Verberk SG de Goede KE Van den Bossche J. Metabolic-Epigenetic Crosstalk in Macrophage Activation: An Updated View. Epigenomics (2019) 11(7):719–21. 10.2217/epi-2019-0073 31150278

[11] Infantino V Iacobazzi V Palmieri F Menga A. ATP-Citrate Lyase is Essential for Macrophage Inflammatory Response. Biochem Biophys Res Commun (2013) 440(1):105–11. 10.1016/j.bbrc.2013.09.037 24051091

[12] Baardman J Verberk SGS van der Velden S Gijbels MJJ van Roomen CPPA Sluimer JC. Macrophage ATP Citrate Lyase Deficiency Stabilizes Atherosclerotic Plaques. Nat Commun (2020) 11(1):1–15. 10.1038/s41467-020-20141-z 33293558 PMC7722882

[13] Lewis AJ Seymour CW Rosengart MR. Current Murine Models of Sepsis. Surg Infect (Larchmt) (2016) 17(4):385–93. 10.1089/sur.2016.021 PMC4960474 27305321

[14] Kratz M Coats Brittney R Hisert Katherine B Hagman D Mutskov V Peris E. Metabolic Dysfunction Drives a Mechanistically Distinct Proinflammatory Phenotype in Adipose Tissue Macrophages. Cell Metab (2014) 20(4):614–25. 10.1016/j.cmet.2014.08.010 PMC4192131 25242226

[15] Lackey DE Olefsky JM. Regulation of Metabolism by the Innate Immune System. Nat Rev Endocrinol (2016) 12(1):15–28. 10.1038/nrendo.2015.189 26553134

[16] Obstfeld AE Sugaru E Thearle M Francisco AM Gayet C Ginsberg HN. C-C Chemokine Receptor 2 (CCR2) Regulates the Hepatic Recruitment of Myeloid Cells That Promote Obesity-Induced Hepatic Steatosis. Diabetes (2010) 59(4):916–25. 10.2337/db09-1403 PMC2844839 20103702

[17] Talukdar S Oh DY Bandyopadhyay G Li D Xu J McNelis J. Neutrophils Mediate Insulin Resistance in Mice Fed a High-Fat Diet Through Secreted Elastase. Nat Med (2012) 18(9):1407–12. 10.1038/nm.2885 PMC3491143 22863787

[18] Tran S Baba I Poupel L Dussaud S Moreau M Gelineau A. Impaired Kupffer Cell Self-Renewal Alters the Liver Response to Lipid Overload During Non-Alcoholic Steatohepatitis. Immunity (2020) 53(3):627–40.e5. 10.1016/j.immuni.2020.06.003 32562600

[19] Liu X Cooper DE Cluntun AA Warmoes MO Zhao S Reid MA. Acetate Production From Glucose and Coupling to Mitochondrial Metabolism in Mammals. Cell (2018) 175(2):502–13.e13. 10.1016/j.cell.2018.08.040 30245009 PMC6173642

[20] Zhao S Torres A Henry RA Trefely S Wallace M Lee JV. ATP-Citrate Lyase Controls a Glucose-to-Acetate Metabolic Switch. Cell Rep (2016) 17(4):1037–52. 10.1016/j.celrep.2016.09.069 PMC5175409 27760311

[21] Fernandez S Viola JM Torres A Wallace M Trefely S Zhao S. Adipocyte ACLY Facilitates Dietary Carbohydrate Handling to Maintain Metabolic Homeostasis in Females. Cell Rep (2019) 27(9):2772–84 e6. 10.1016/j.celrep.2019.04.112 31141698 PMC6608748

[22] Gao X Lin SH Ren F Li JT Chen JJ Yao CB. Acetate Functions as an Epigenetic Metabolite to Promote Lipid Synthesis Under Hypoxia. Nat Commun (2016) 7:11960. 10.1038/ncomms11960 27357947 PMC4931325

[23] Leone RD Zhao L Englert JM Sun I-M Oh M-H Sun I-H. Glutamine Blockade Induces Divergent Metabolic Programs to Overcome Tumor Immune Evasion. Science (2019) 366(6468):1013–21. 10.1126/science.aav2588 PMC7023461 31699883

[24] Kendrick SF O’Boyle G Mann J Zeybel M Palmer J Jones DE. Acetate, the Key Modulator of Inflammatory Responses in Acute Alcoholic Hepatitis. Hepatology (2010) 51(6):1988–97. 10.1002/hep.23572 20232292

[25] Zhang Z La Placa D Nguyen T Kujawski M Le K Li L. CEACAM1 Regulates the IL-6 Mediated Fever Response to LPS Through the RP105 Receptor in Murine Monocytes. BMC Immunol (2019) 20(1):7. 10.1186/s12865-019-0287-y 30674283 PMC6345024

[26] Zhang Z Wang M Zhang Y Zhang Y Bartkuhn M Markmann M. Uropathogenic Escherichia Coli Virulence Factor α Hemolysin Reduces Histone Acetylation to Inhibit Expression of Pro-Inflammatory Cytokine Genes. J Infect Dis (2021) 223(6):1041–51. 10.1093/infdis/jiab018 33453118

[27] Wang C Zhu X Cui Y Miao H Xu Y Xiong X. Serum Proteome-Wide Identified ATP Citrate Lyase as a Novel Informative Diagnostic and Prognostic Biomarker in Pediatric Sepsis: A Pilot Study. Immun Inflammation Dis (2020). 10.1002/iid3.399 PMC8127565 33378581

[28] Benoit M Desnues B Mege JL. Macrophage Polarization in Bacterial Infections. J Immunol (2008) 181(6):3733–9. 10.4049/jimmunol.181.6.3733 18768823

[29] Brini E Ruffini F Bergami A Brambilla E Dati G Greco B. Administration of a Monomeric CCL2 Variant to EAE Mice Inhibits Inflammatory Cell Recruitment and Protects From Demyelination and Axonal Loss. J Neuroimmunol (2009) 209(1-2):33–9. 10.1016/j.jneuroim.2009.01.022 19232440

[30] Ummenthum K Peferoen LA Finardi A Baker D Pryce G Mantovani A. Pentraxin-3 is Upregulated in the Central Nervous System During MS and EAE, But Does Not Modulate Experimental Neurological Disease. Eur J Immunol (2016) 46(3):701–11. 10.1002/eji.201545950 26576501

[31] Baardman J Verberk SGS Prange KHM van Weeghel M van der Velden S Ryan DG. A Defective Pentose Phosphate Pathway Reduces Inflammatory Macrophage Responses During Hypercholesterolemia. Cell Rep (2018) 25(8):2044–52.e5. 10.1016/j.celrep.2018.10.092 30463003

[32] Zhou Y Zhou B Pache L Chang M Khodabakhshi AH Tanaseichuk O. Metascape Provides a Biologist-Oriented Resource for the Analysis of Systems-Level Datasets. Nat Commun (2019) 10(1):1523. 10.1038/s41467-019-09234-6 30944313 PMC6447622

[33] Hussaarts L Garcia-Tardon N van Beek L Heemskerk MM Haeberlein S van der Zon GC. Chronic Helminth Infection and Helminth-Derived Egg Antigens Promote Adipose Tissue M2 Macrophages and Improve Insulin Sensitivity in Obese Mice. FASEB J (2015) 29(7):3027–39. 10.1096/fj.14-266239 25852044

[34] van der Zande HJP Gonzalez MA de Ruiter K Wilbers RHP Garcia-Tardon N van Huizen M. The Helminth Glycoprotein Omega-1 Improves Metabolic Homeostasis in Obese Mice Through Type 2 Immunity-Independent Inhibition of Food Intake. FASEB J (2021) 35(2):e21331. 10.1101/2020.07.03.186254 33476078 PMC7898285

