# Supplementary material for: Myeloid ATP Citrate Lyase Regulates Macrophage Inflammatory Responses In Vitro Without Altering Inflammatory Disease Outcomes
Source: Front Immunol. 2021 Apr 26;12:669920. doi: 10.3389/fimmu.2021.669920 (PMC8107722; doi:10.3389/fimmu.2021.669920)
Supplement: Supplementary file 1 [file DataSheet_1.pdf]

Supplementary information for:

Myeloid ATP citrate lyase regulates macrophage inflammatory responses *in vitro* without altering inflammatory disease outcomes

Sanne G.S. Verberk[1,#], Hendrik J.P. van der Zande[2,#], Jeroen Baardman[3], Kyra E. de Goede[1], Karl J. Harber[1,3], Eelco D. Keuning[1], Joost M. Lambooij[2], Frank Otto[2], Anna Zawistowska-Deniziak[2,4], Helga E. de Vries[1], Menno P.J. de Winther[3], Bruno Guigas[2], Jan Van den Bossche[1]*

[1]Department of Molecular Cell Biology and Immunology, Amsterdam Cardiovascular Sciences, Amsterdam Gastroenterology Endocrinology Metabolism, Amsterdam UMC, Vrije Universiteit Amsterdam, Amsterdam, Netherlands
[2]Department of Parasitology, Leiden University Medical Center, Leiden, Netherlands
[3]Department of Medical Biochemistry, Experimental Vascular Biology, Amsterdam Cardiovascular Sciences, Amsterdam UMC, University of Amsterdam, Amsterdam, Netherlands
[4]Witold Stefański Institute of Parasitology, Polish Academy of Sciences, Warsaw, Poland

[#]Contributed equally
*Corresponding Author:
Jan Van den Bossche
j.vandenbossche@amsterdamumc.nl

Contents:

Supplementary Figure 1
Supplementary Figure 2
Supplementary Figure 3
Supplementary Table 1 - Cell abundance in blood and peritoneal lavage
Supplementary Table 2 - List of primers used for the determination of gene expression levels
Supplementary Table 3 - List of used antibodies

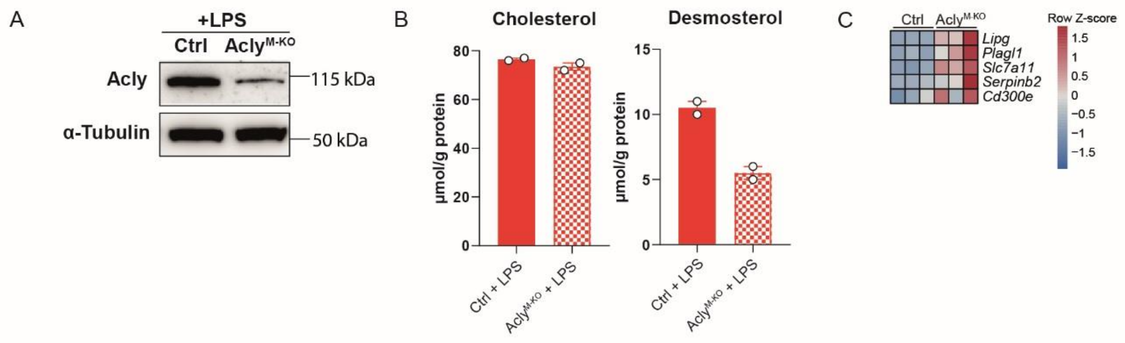

Supplementary Figure 1 (**A**) Protein levels of Acly in 24-hour LPS-activated BMDMs, assessed by immunoblotting analysis. (**B**) Cholesterol and desmosterol levels in 24-hour LPS-activated BMDMs. (**C**) Significantly regulated LPS-induced genes in unstimulated BMDMs.

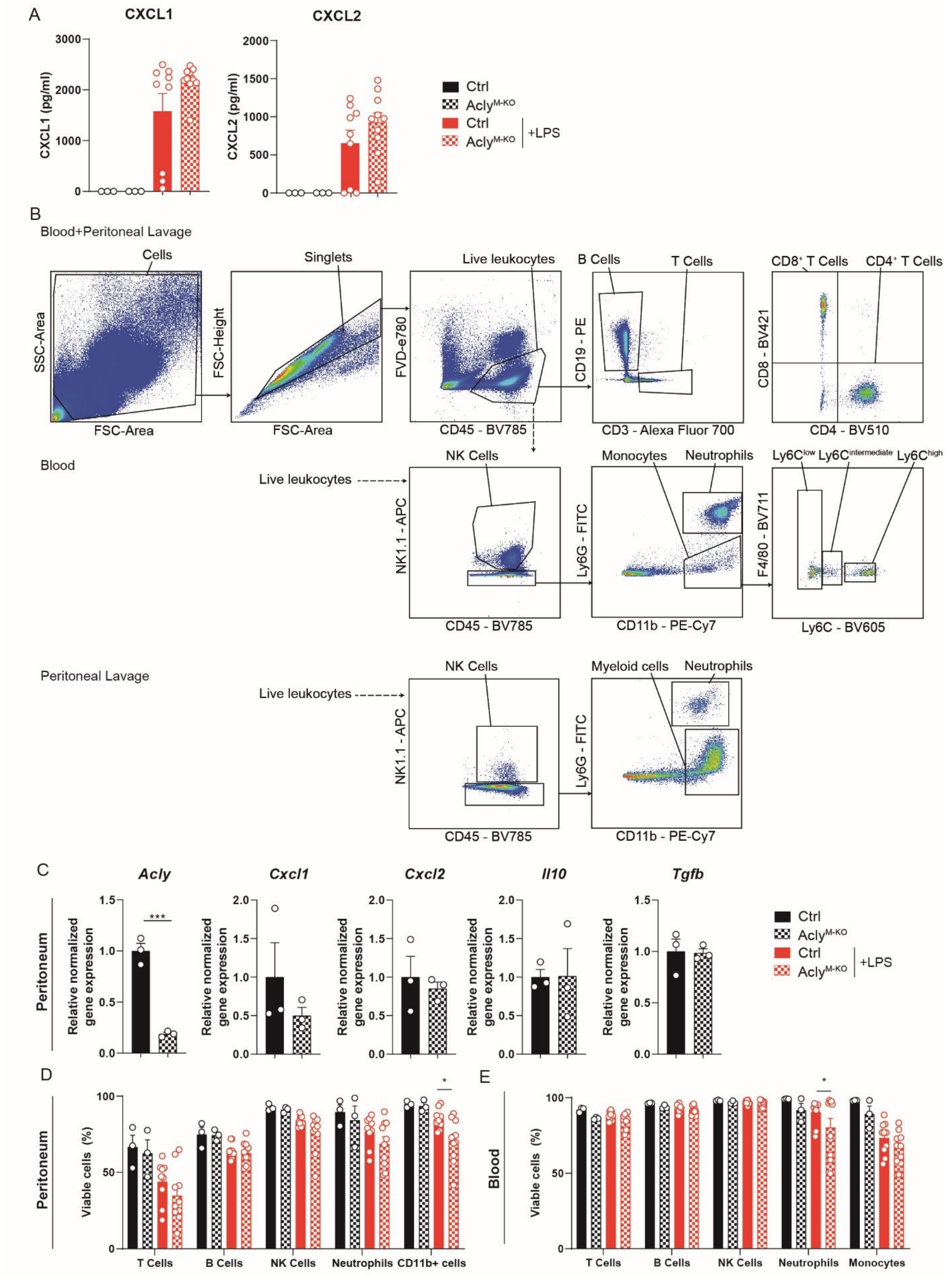

Supplementary Figure 2. (A) Levels of CXCL1 and CXCL2 in peritoneal lavage. (**B**) Flow cytometry gating strategy for analysis of blood and peritoneal B cells, T cells, $CD4^+$/$CD8^+$ T cells, NK cells, Monocytes/Myeloid cells, and Neutrophils. (**C**, **D**) Viability of cell subsets as defined by flow cytometry in peritoneum (**C**) and blood (**D**). **E**) Gene expression on peritoneal exudate cells atbaseline. Values represent mean±SEM (n=3/3/10/10 (Ctrl vehicle/KO vehicle/Ctrl LPS/KO LPS)). *P<0.05 by ordinary Two-way ANOVA with Sidak's post hoc test for multiple comparisons.

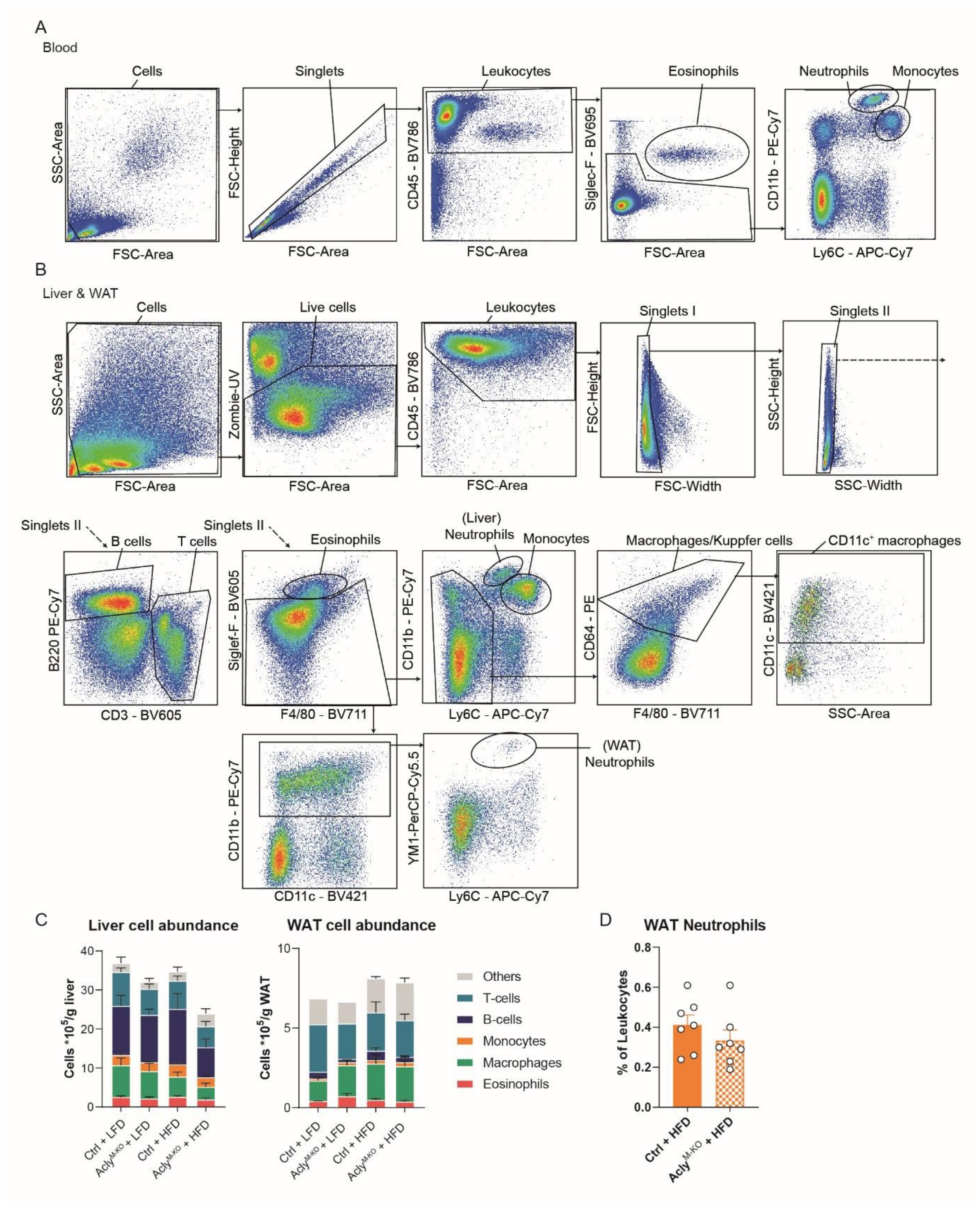

Supplementary Figure 2. (**A,B**) Flow cytometry gating for eosinophils, neutrophils and monocytes in blood (**A**) and for eosinophils, neutophils, monocytes, macrophages (Kuppfer cells in liver), CD11c$^+$ macrophages, B cells and T cells in liver and WAT. (**C**) Flow cytometric detmination of immune cell composition in liver and WAT per gram of tissue. (**D**) Neutrophil abundance in WAT upon HFD feeding. Values represent mean ± SEM (n=3/3/6/7 (Ctrl LFD, KO LFD, Ctrl HFD, KO HFD))

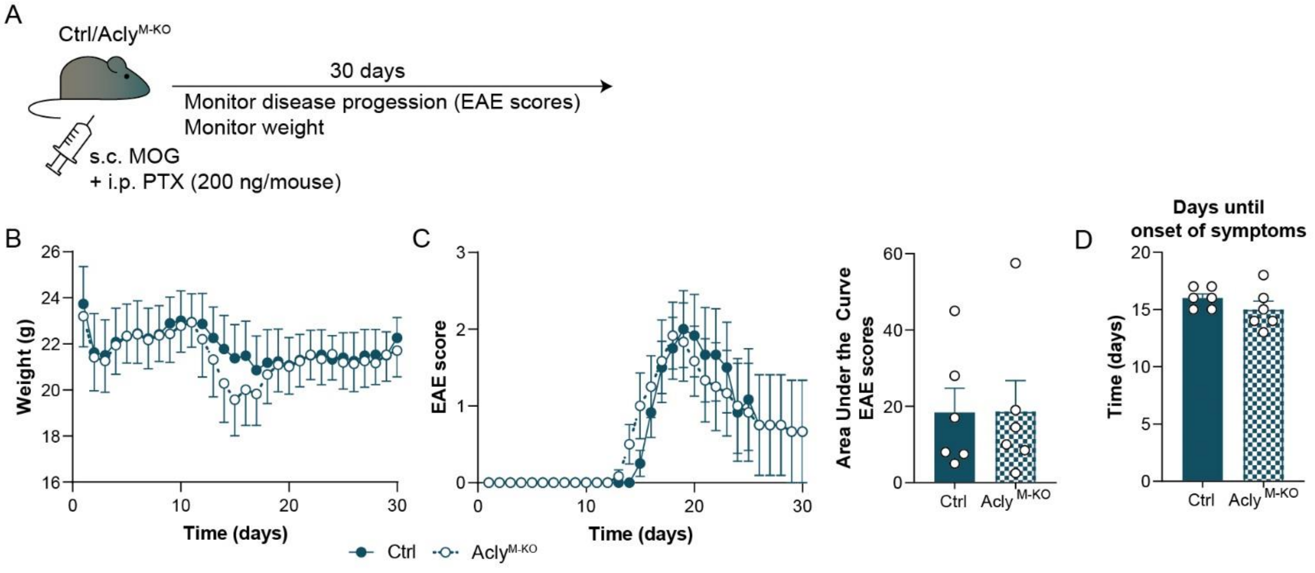

Supplementary Figure 3. Experimental autoimmune encephalitis. (**A**) Control and Acly$^{M\text{-}KO}$ mice were injected subcutaneously with a myelin oligodendrocyte (MOG)-solution and intraperitoneally wth pertussis toxin (PTX). (**B**) Weight progression during the course of 30 days in control and Acly$^{M\text{-}KO}$ mice. (**C**) EAE scoring during the course of 30 days and derived area under the curve (AUC) in control and Acly$^{M\text{-}KO}$ mice. (**D**) Days until onset of symptoms in control and Acly$^{M\text{-}KO}$ mice. Values represent mean ± SEM (n=6/6 (ctrl/Acly$^{M\text{-}KO}$)

Supplementary Table 1. Cell abundance in blood and peritoneal lavage (PL), accompanying Figure 2E,F

	**Ctrl**		**Acly^{M-KO}**		**Ctrl + LPS**		**ACLY^{M-KO} + LPS**	
**PL**	**Average**	**SD**	**Average**	**SD**	**Average**	**SD**	**Average**	**SD**
NK Cells	1.8	0.5	1.3	0.2	1.3	0.5	1.2	0.3
Neutrophils	0.4	0.2	5.6 *	4.0	2.0	1.3	0.8[#]	2.6
CD11b+ cells	50.4	8.0	44.0	17.2	42.1	13.5	24.1 *	9.7
B Cells	35.0	7.8	36.1	14.4	42.4	10.3	61.5	11.4
T Cells	5.2	0.8	3.1	0.6	4.3	0.9	3.4	0.4
- CD8+ T Cells	12.3	1.4	9.3	3.1	10.6	3.5	8.8	1.6
- CD4+ T Cells	67.4	8.3	55.7	18.4	69.0	6.0	75.2	7.6
(n=)	3		3		9		10	
	**Ctrl**		**Acly^{M-KO}**		**Ctrl + LPS**		**ACLY^{M-KO} + LPS**	
**Blood**	**Average**	**SD**	**Average**	**SD**	**Average**	**SD**	**Average**	**SD**
NK Cells	4.7	1.2	4.0	0.2	14.4	5.6	21.2	7.3
Neutrophils	10.0	5.3	5.1	3.1	13.3	4.6	16.1[#]	12.4
Monocytes	10.4	0.5	9.1	3.1	2.1	1.1	1.9	0.8
B Cells	57.7	6.4	62.3	3.8	51.2	7.8	42.8	8.5
T Cells	13.8	4.7	15.7	3.1	13.8	5.0	12.7	4.8
- CD8+ T Cells	48.4	2.4	47.7	1.1	51.4	2.0	54.9	4.0
- CD4+ T Cells	49.2	2.1	49.4	0.9	45.5	2.4	41.8	5.3
(n=)	3		3		10		10	

1 outlier detected with ROUT's outlier test (Graphpad Prism 8.2.1)
* P<0.05, significant difference between Ctrl and Acly^{M-KO} from same stimulation

Supplementary Table 2. List of primer sequences

Marker	Forward primer	Reverse primer
**Endotoxin-induced peritonitis model**		
Rplp0	GGACCCGAGAAGACCTCCTT	GCACATCACTCAGAATTTCAATGG
Ppia	TTCCTCCTTTCACAGAATTATTCCA	CCGCCAGTGCCATTATGG
Il1b	AAAGAATCTATACCTGTCCTGTGTAATGAAA	GGTATTGCTTGGGATCCACACT
Il6	GCTACCAAACTGGATATAATCAGGAAA	CTTGTTATCTTTTAAGTTGTTCTTCATGTACTC
Il10	TTTGAATTCCCTGGGTGAGAA	CTCCACTGCCTTGCTCTTATTTTC
Tnf	CATCTTCTCAAAATTCGAGTGACAA	TGGGAGTAGACAAGGTACAACCC
Il12b	GGTGCAAAGAAACATGGACTTG	CACATGTCACTGCCCGAGAGT
Acly	CCCCAAGATTCAGTCCCAAGT	GCCTTGGTATGTCGGCTGAA
Cxcl1	ACCCAAACCGAAGTCATAGCC	AGACAGGTGCCATCAGAGC
Cxcl2	CATCCAGAGCTTGAGTGTGAC	CTTTGGTTCTTCCGTTGAGGG
Tgfb	GTCACTGGAGTTGTACGGCA	AGCCCTGTATTCCGTCTCCT
**Obesity model**		
Rplp0	TCTGGAGGGTGTCCGCAACG	GCCAGGACGCGCTTGTACCC
Adgre	CTTTGGCTATGGGCTTCCAGTC	GCAAGGAGGACAGAGTTTATCGTG
Itgax	GCCACCAACCCTTCCTGGCTG	TTGGACACTCCTGCTGTGCAGTTG
Ccl2	TCAGCCAGATGCAGTTAACGCCC	GCTTCTTTGGGACACCTGCTGCT
Tnf	GTCCCCAAAGGGATGAGAAG	CACTTGGTGGTTTGCTACGA

Supplementary Table 3. List of antibodies used

Marker	Fluorochrome	Clone	Supplier	Catalog Number
**Endotoxin-induced peritonitis model**				
**FVD**	e780		eBioscience	65-0865-14
**CD45**	BV785	30-F11	Biolegend	103149
**CD19**	PE	6D5	Biolegend	115507
**CD3**	Alexa Fluor 700	17A2	Biolegend	100215
**CD8a**	BV421	53-6.7	Biolegend	100753
**CD4**	BV510	RM4-5	Biolegend	100553
**NK1.1**	APC	PK136	Biolegend	108709
**Ly6G**	FITC	1A8	Biolegend	127605
**CD11b**	BV650	M1/70	Biolegend	101239
**F4/80**	BV711	BM8	Biolegend	123147
**Ly6C**	BV605	HK1.4	Biolegend	128035
**Obesity model**				
**Zombie**	UV		Invitrogen	423107
**CD45**	BV785	30-F11	Biolegend	103149
**Siglec-F**	BV605	E50-2440	BD Biosciences	740388
**CD11b**	PE-Cy7	M1/70	eBioscience	25-0112
**CD11b**	FITC	M1/70	eBioscience	11-0112
**Ly6C**	APC-Cy7	HK1.4	Biolegend	128026
**CD64**	PE	X54-5/7.1	Biolegend	139304
**F4/80**	BV711	BM8	Biolegend	123147
**CD11c**	BV421	N418	Biolegend	117330
**CD11c**	FITC	HL3	BD Biosciences	553801
**B220**	PE-Cy7	RA3-6B2	Biolegend	103221
**CD3**	BV605	17A2	Biolegend	100237
**GR-1**	FITC	RB6-8C5	BD Biosciences	553126
**NK1.1**	FITC	PK136	eBioscience	11-5941
